# Opportunistic computed tomography assessment of bone, muscle, and adipose tissue: evidence, limitations, and clinical translation

**DOI:** 10.3389/fmed.2026.1923817

**Published:** 2026-09-28

**Authors:** Ze Liu, Hao Ge

**Affiliations:** 1 Department of Nuclear Medicine, Ningbo Hangzhou Bay Hospital (Ningbo Branch of Renji Hospital, Shanghai Jiao Tong University School of Medicine), Ningbo, Zhejiang, China; 2 Department of Nuclear Medicine, Taizhou First People’s Hospital, Taizhou, Zhejiang, China

**Keywords:** body-composition, myosteatosis, opportunistic computed tomography, osteoporosis, sarcopenia, vertebral attenuation, vertebral fracture, visceral adiposity

## Abstract

Routine body computed tomography (CT) contains quantitative information beyond the organ or disease for which the examination was requested. When technically suitable images have already been acquired, vertebral trabecular attenuation, vertebral fractures, skeletal muscle cross-sectional area and attenuation, and visceral and subcutaneous adipose tissue can be assessed without an additional scan or additional radiation exposure. This narrative review separates analytical validity, clinical association and prediction, clinical utility, demonstrated patient benefit, and implementation readiness. Vertebral fracture detection and protocol-specific vertebral attenuation currently have the most direct connection to established osteoporosis pathways, whereas muscle measures provide mainly disease-specific risk information and adipose, longitudinal, combined, and automated applications remain more heterogeneous. CT muscle area is a surrogate for muscle quantity rather than a direct measurement of muscle mass, and CT alone does not diagnose sarcopenia. Visceral adipose tissue area, subcutaneous adipose tissue area, the visceral-to-subcutaneous adipose tissue ratio, adipose attenuation, and longitudinal fat loss are distinct constructs and do not provide CT-only diagnoses of metabolic syndrome, malnutrition, cachexia, or sarcopenic obesity. Recent automated systems show strong technical performance, including external and prospective workflow evaluations, but evidence that routine automated reporting improves patient outcomes remains limited. Combined bone-muscle-adipose assessment is biologically plausible but should currently be regarded as hypothesis-generating unless incremental predictive or clinical value is demonstrated. Translation therefore requires protocol-aware measurement, repeatability assessment, visible quality control, explicit responsibility for verification and follow-up, and prospective evaluation of patient benefit, workload, equity, and cost-effectiveness.

## 1 Introduction

Routine body computed tomography (CT) contains quantitative information beyond the primary clinical indication (1–5). Thoracic, abdominal, pelvic, oncologic, trauma, vascular, and selected hybrid examinations often include vertebral bodies, skeletal muscle, visceral adipose tissue (VAT), and subcutaneous adipose tissue (SAT) (1–4). When image quality, reconstruction, field of view, and anatomic coverage are suitable, these structures can be assessed from images that have already been acquired, without an additional examination or additional radiation exposure (1–5). Automated extraction, structured display, and reporting may further support incorporation of these measurements into imaging workflows (3, 4, 6–10).

Among these applications, opportunistic assessment of bone has the clearest connection to an established clinical pathway (5, 9, 11–14). Vertebral trabecular attenuation can provide protocol-dependent information associated with bone mineral status and fracture risk, but it remains an imaging risk marker rather than a dual-energy x-ray absorptiometry (DXA)-equivalent diagnosis (8, 12, 13, 15–21). A visually confirmed low-trauma vertebral fracture may constitute a clinically consequential fracture history and can connect directly to an established osteoporosis pathway (11, 14, 22). Low attenuation and vertebral fracture should therefore be distinguished, and fracture interpretation should consider trauma, malignancy, infection, congenital or degenerative deformity, and other mimics (11, 20, 22).

Foundational single-slice body-composition studies established the rationale for using standardized abdominal cross-sectional area as a surrogate for broader muscle quantity (23, 24). Within CT-specific applications, CT provides quantitative skeletal muscle cross-sectional area (2, 6, 7, 24) and attenuation-based measures of tissue composition (2, 25, 26). Related automated body-composition work has quantified VAT/SAT longitudinally (27), while multi-biomarker and dedicated muscle pipelines have added outcome prediction and automated muscle measurement (28, 29). Cross-sectional area should still be interpreted as a surrogate for muscle quantity rather than a direct measurement of muscle mass (23, 24, 30), whereas attenuation provides protocol-sensitive information related to tissue composition (25, 31–33). Selected oncology cohort studies report prognostic or nutritional associations (34–38), while cancer-cachexia consensus and review literature provide clinical context for interpreting muscle depletion (39, 40). Disease-specific trauma and liver studies provide additional associations (41–43). Their values are influenced by anatomic level, acquisition technique, contrast phase, segmentation method, normalization, and population-specific thresholds (2, 6, 7, 25, 31–33). CT alone does not establish sarcopenia, which requires the clinical elements specified by the applicable consensus framework (44–46).

The European Society for Clinical Nutrition and Metabolism/European Association for the Study of Obesity (ESPEN/EASO) sarcopenic-obesity consensus underscores that adiposity should be interpreted together with muscle status rather than used as a CT-only diagnosis (47). CT can quantify and separate visceral and subcutaneous adipose compartments (6, 7, 24, 27, 48). Longitudinal adipose change can be evaluated when technically comparable serial examinations are available (27). VAT area and SAT area quantify distinct compartments, whereas the VAT/SAT ratio is a relative distribution metric (27, 28). Adipose attenuation is a separate radiodensity measure (48), and tissue loss describes longitudinal change rather than a baseline phenotype (27). Contrast-phase effects are considered separately below (32, 48). CT-derived adipose measurements can provide cohort-specific risk or body-composition information (27, 28). These measures should therefore be interpreted as biomarkers or body-composition descriptors rather than as standalone clinical diagnoses (28, 39, 40, 47). In the Wisconsin screening cohort, the VAT/SAT ratio functioned as a risk biomarker rather than a CT diagnostic definition of metabolic syndrome (28), and CT-defined abnormal body-composition phenotypes can be discordant with conventional nutrition-screening classifications in oncology (38).

Bone and skeletal muscle interact mechanically and metabolically, and osteosarcopenia literature also describes fat-related pathways linking these compartments (49). The ESPEN/EASO sarcopenic-obesity consensus separately addresses the clinically relevant coexistence of excess adiposity and impaired muscle mass/function (47). The 2025 Asian Working Group for Sarcopenia (AWGS) update also emphasizes skeletal-muscle cross-talk with bone, adipose tissue, and immune systems (44). Osteosarcopenia and sarcopenic obesity are therefore related but distinct clinical constructs (47, 49). A recent integrated CT bone-muscle study remained cross-sectional and did not test incremental predictive or patient-outcome benefit of a combined model (30).

Accordingly, this review treats vertebral attenuation as an imaging risk marker rather than a DXA-equivalent diagnosis (12, 20), skeletal muscle CT measures as imaging biomarkers that require the applicable clinical sarcopenia assessment (45, 46), and adipose findings as body-composition measures rather than CT-only sarcopenic-obesity diagnoses (47). A visually confirmed fragility-compatible vertebral fracture is treated separately because it can enter an established osteoporosis pathway (11, 14). The review distinguishes analytical validity, clinical association and prediction, clinical utility, demonstrated patient benefit, and implementation evidence so that technical performance is not conflated with clinical effect. The overall role of opportunistic CT is therefore framed primarily as risk enrichment and clinical triage within appropriately validated pathways rather than as a replacement for established diagnostic assessment (3, 5, 9).

## 2 Review scope and search strategy

### 2.1 Narrative search strategy

This article is a narrative review rather than a systematic review or meta-analysis. It was structured and critically appraised with reference to the six quality domains of the Scale for the Assessment of Narrative Review Articles (SANRA): justification of the review, statement of aims, description of the literature search, referencing, scientific reasoning, and presentation of relevant endpoint data (50). SANRA was used as a quality-assessment framework rather than as a reporting guideline. For expert-opinion, consensus/guideline, policy, and other textual-evidence sources, interpretation additionally considered the source-specific principles of the updated textual-evidence framework described by McArthur et al. (51); those tools were not applied as substitutes for design-specific appraisal of primary quantitative studies.

Relevant literature was identified through targeted searches of PubMed/MEDLINE and Google Scholar covering database inception through 25 August 2026, supplemented by publisher websites and backward and forward citation tracking from systematic reviews, narrative reviews, consensus statements, expert recommendations, and primary studies. The review was designed to identify representative evidence addressing measurement, association or prediction, clinical utility, implementation, and patient outcomes rather than to exhaustively retrieve all eligible publications. Representative PubMed search strings included: (“opportunistic CT” OR “opportunistic computed tomography”) AND (osteoporosis OR “vertebral fracture” OR “bone mineral density”); (CT OR “computed tomography”) AND (“skeletal muscle” OR sarcopenia OR myosteatosis) AND (segmentation OR “artificial intelligence” OR “deep learning”); and (CT OR “computed tomography”) AND (“visceral adipose” OR “subcutaneous adipose” OR “body composition”) AND (segmentation OR contrast OR longitudinal). Additional focused searches addressed photon-counting CT, repeatability, implementation, reimbursement, and quality control.

Publications were prioritized when they evaluated measurement validity or repeatability, compared CT-derived measures with an established reference method, examined clinically relevant association or prediction, provided systematic synthesis or consensus guidance, externally or multicentrically validated automated methods, or addressed prospective or real-world implementation. Foundational studies were retained when they established widely used measurement methods or concepts, while recent independent evidence was added to reduce reliance on older or single-group literature. Publications arising from related institutions or related screening cohorts were treated as potentially non-independent when overlap could reasonably be suspected rather than being counted as independent replication.

Organ-specific incidental findings, hepatic steatosis, vascular calcification, and broad cardiometabolic risk models were excluded unless they directly informed adipose interpretation, implementation, or the three-compartment framework. The narrative search did not retain a contemporaneous screening log; therefore, the number of records considered cannot be reconstructed reliably and was not estimated retrospectively. The synthesis includes 67 cited sources. No protocol was registered, and no systematic-review flow diagram, duplicate independent screening, formal study-by-study risk-of-bias assessment, or quantitative synthesis was undertaken. Study identification and selection were therefore not exhaustive and may have been affected by selection, citation, and publication bias. The conclusions are presented as a structured narrative synthesis rather than as formal evidence grades or professional-society recommendations.

### 2.2 Framework for narrative evidence interpretation

For clarity, this review treats six questions separately: analytical validity, clinical association, prediction, clinical utility, demonstrated patient benefit, and implementation readiness. Analytical validity asks whether a measurement can be obtained accurately and reproducibly under specified technical conditions. Clinical association asks whether the measurement agrees with a reference standard or is associated with a clinically relevant state or outcome. Prediction asks whether a measurement or model discriminates or calibrates future risk in an appropriately evaluated cohort. Clinical utility asks whether use of the information changes management, whereas patient benefit asks whether such changes improve outcomes important to patients. Implementation readiness concerns safe and sustainable integration into routine workflow. Strong segmentation performance or a retrospective association should therefore not be interpreted as proof that reporting the biomarker changes care or improves outcomes.

Evidence maturity was judged narratively from study design, external validation, cohort independence, consistency, prospective or real-world evaluation, and proximity to an established clinical pathway. Table 1 is an author-developed synthesis intended to distinguish relatively mature evidence from emerging or hypothesis-generating applications. It is not a formal evidence-grading framework, guideline, or professional-society classification. This conceptual hierarchy is summarized in Figure 1.

**Table 1:** Narrative summary of evidence maturity, potential roles, and boundaries of opportunistic CT biomarkers.

Biomarker or application	Evidence maturity and supporting evidence	Potential current role	Principal boundary
Vertebral trabecular HU attenuation	Relatively mature for protocol-specific risk enrichment; supported by foundational studies, external validation, systematic review, large-scale automation, and contrast-effect studies (8, 12, 13, 15–21, 31).	Identify patients who may merit DXA or clinical fracture-risk assessment within a locally validated pathway.	Protocol-, level-, contrast-, scanner-, reconstruction-, ROI-, calibration-, and population-dependent; not equivalent to a DXA T-score or universal osteoporosis diagnosis.
Prevalent vertebral fracture	Most direct clinical pathway among the reviewed applications; visual assessment, underreporting, grading, and downstream osteoporosis pathways are established concepts (9, 11, 14, 22).	Communicate a confirmed clinically consequential vertebral fracture and consider an established osteoporosis pathway when compatible with fragility fracture.	Requires visual confirmation and differentiation from trauma, tumor, infection, congenital deformity, and degenerative mimics.
L3 muscle area/skeletal muscle index and muscle attenuation	Technically well validated by CT-specific internal, external, dual-center, and prospective automated workflows (2, 6, 7, 10, 24); foundational single-slice rationale also includes modality-independent body-composition work (23), while clinical associations and thresholds remain disease- and method-specific (34–38, 41–43).	Disease-specific prognostic, nutritional, or frailty risk enrichment and support for further clinical assessment.	Cross-sectional area is a surrogate for muscle quantity; CT alone does not diagnose sarcopenia; attenuation is contrast-phase sensitive.
VAT/SAT measures and adipose attenuation	CT can segment VAT and SAT with automated methods (6, 7, 10, 27); contrast phase and segmentation method can alter measurements (32, 48), and risk associations are cohort-specific (28).	Describe regional adipose distribution or technically comparable change when relevant to the clinical context.	No universal CT-only metabolic, nutritional, cachexia, or sarcopenic-obesity threshold; contrast phase and segmentation method can alter values.
Spectral and photon-counting CT bone measures	Emerging technical evidence. Earlier dual-layer spectral scout work showed feasibility, and prospective photon-counting detector CT spectral-localizer work showed agreement with DXA-derived measures (64, 65).	Research and selected locally validated opportunistic screening workflows.	Platform-, calibration-, and vendor-dependent; broad external validation and patient-benefit evidence remain limited.
Longitudinal muscle/adipose change	Emerging; serial measurement is feasible (27, 29, 36), but interpretation requires precision testing and method-specific error limits (62, 63).	Research and selected disease-specific monitoring when acquisition and analysis are comparable.	Observed change should exceed method-specific measurement error or least significant/minimum detectable change before being interpreted as biological progression or response.
Integrated scores and automated dashboards	Hypothesis-generating to emerging. Retrospective risk models, integrated cross-sectional assessment, and prospective automated workflows exist, but patient-benefit evidence remains limited (5, 9, 10, 30, 57, 58).	Local validation, controlled pilots, and hypothesis generation.	No universal CT-only syndrome definition; incremental predictive value, clinical utility, governance, equity, and cost-effectiveness require prospective evaluation.

**Figure 1:**
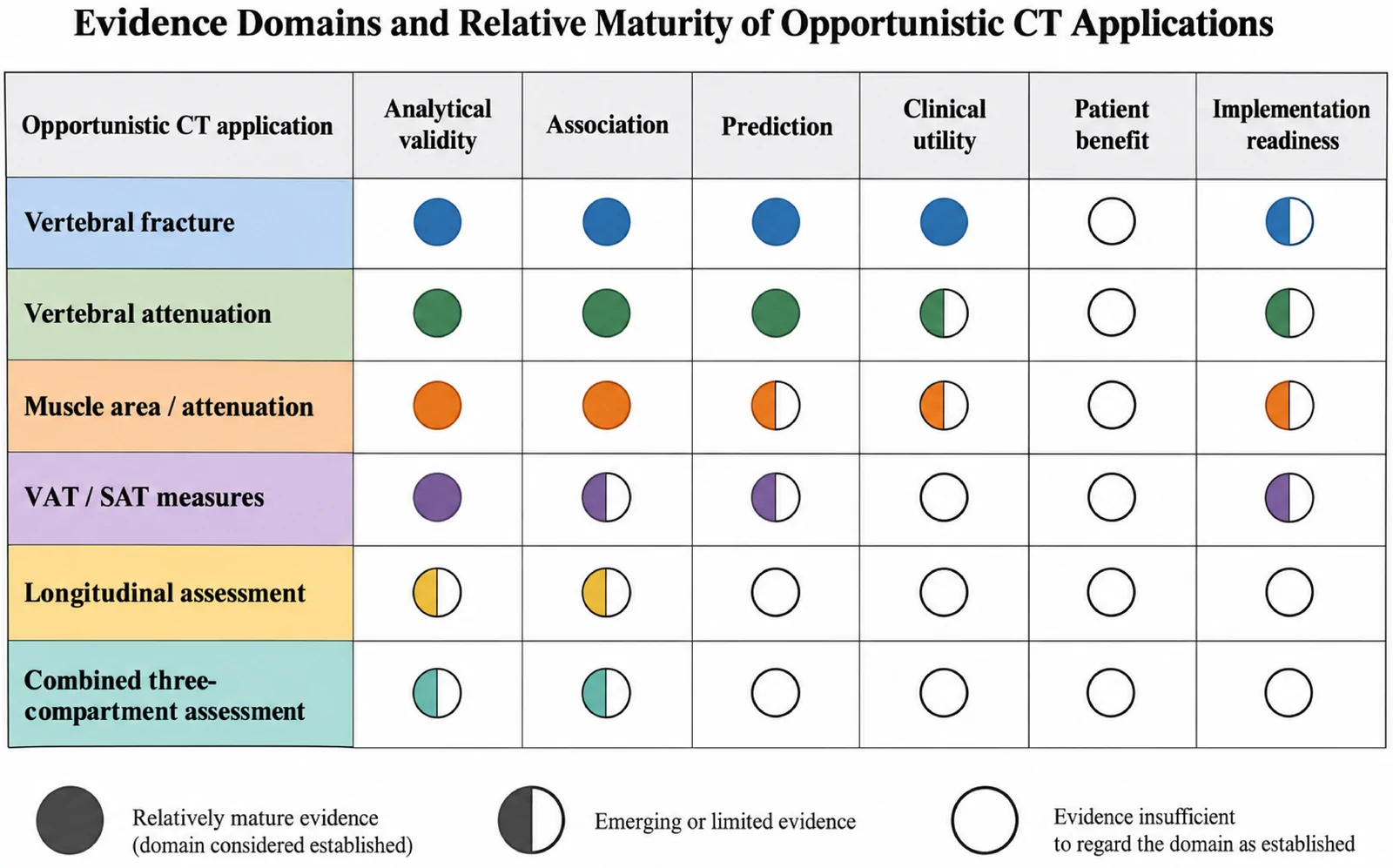
Evidence domains and relative maturity of opportunistic CT applications. This author-proposed conceptual synthesis separates analytical validity, clinical association, prediction, clinical utility, patient benefit, and implementation readiness; it is not a formal evidence-grading system. Colors distinguish applications, whereas filled, half-filled, and open circles indicate relatively mature, emerging or limited, and currently insufficient evidence, respectively, within the literature reviewed. These categories are relative and should not be interpreted as proof of universal thresholds, demonstrated patient benefit, or routine implementation readiness. Vertebral fracture detection and protocol-specific vertebral attenuation currently have the most direct clinical pathways (5, 9, 11, 12, 14). Muscle evidence is predominantly technical or disease-specific (2, 6, 7, 10, 34–38, 41–43), while longitudinal interpretation requires explicit precision and measurement-error assessment (62, 63). The combined three-compartment row represents an author-proposed, hypothesis-generating synthesis of bone, muscle, and adipose assessment rather than a validated three-compartment diagnostic or prognostic model; currently available direct integrated CT evidence remains predominantly cross-sectional (30). Automated applications likewise require further outcome-based validation (9, 10).

## 3 Bone compartment: vertebral attenuation and vertebral fracture

### 3.1 Vertebral trabecular attenuation

Trabecular attenuation measured within a vertebral body, most commonly L1 when available, is one of the most extensively studied opportunistic CT bone measurements (12, 13, 15–20). Foundational abdominal CT and CT-colonography studies established associations between vertebral attenuation and DXA-derived bone mineral status (13, 15), whereas external validation and systematic review showed meaningful but heterogeneous performance across populations, protocols, and reference standards (12, 17, 18). Westerhoff et al. extended automated assessment to 538,946 noncontrast CT examinations from 283,499 patients, acquired using 43 scanner models and six tube voltages, with greater than 99% agreement for a sampled set of automated three-dimensional region-of-interest (ROI) placements (8). That study also showed substantial effects of tube voltage and scanner model and explicitly noted that its prevalence-derived thresholds were not validated against DXA-based diagnosis, so its values should not be transferred to other ROI methods without validation (8).

For manual L1 measurement, an ovoid ROI is commonly placed within anterior trabecular bone while avoiding cortex, focal abnormalities or lesions, artifacts, and the midvertebral high-density zone (16). Automated 3D methods use different placement rules; for example, Westerhoff et al. used a spherical ROI constrained within the segmented vertebral body and anterior to the basivertebral foramen, with automated consistency checks (8). The vertebral level, ROI method, and reason for using an alternative vertebra should be documented because attenuation differs by vertebral level and ROI placement (8, 16). If L1 is unsuitable, an adjacent vertebra may be measured, but the level must be reported and interpretation should be level- and method-specific rather than assumed interchangeable (8, 16). Automated systems may improve throughput (8), while implementation frameworks retain review of the selected vertebra, measurement region, and failure or consistency flags by an imaging professional (9).

A vertebral Hounsfield unit (HU) value is not interchangeable with a DXA T-score or calibrated quantitative CT measurement (12, 20). DXA itself requires standardized acquisition, analysis, interpretation, and reporting for valid diagnostic classification and monitoring (52). Intravenous contrast alters vertebral attenuation, but the magnitude depends on contrast phase, protocol, scanner, and analysis method (19, 21, 31). Pickhardt et al. reported an average increase of approximately 11 HU at L1 under their paired abdominal-CT protocol (19), whereas an independent four-phase study by Boutin et al. observed a mean portal-phase increase of 33.1 HU at L4 cancellous bone and substantial intersubject variation (31). More recent multiphase work likewise showed contrast-related shifts in CT-derived bone-density classifications (21). A single fixed correction factor should therefore not be treated as universal; vertebral level, kilovolt peak (kVp), contrast status or phase, reconstruction, and measurement method should accompany the reported value (8, 19, 21, 31).

Low-dose chest CT can support bone fragility assessment when included vertebrae and acquisition methods have been validated (53, 54). Vertebral attenuation on low-dose chest CT has been associated with DXA-derived bone status and vertebral fracture, and automated systems have shown favorable technical performance in lung-screening cohorts (53, 54). Disease-specific thresholds, such as those proposed in diabetes cohorts, should be described as cohort-specific screening triggers rather than generalized diagnostic criteria (55).

### 3.2 Vertebral fracture and fracture-risk enrichment

A prevalent vertebral fracture has a different clinical meaning from low vertebral attenuation (11, 14, 22, 56). Low attenuation is a protocol-dependent risk marker, whereas a confirmed low-trauma vertebral fracture may constitute a clinically consequential fracture history and can connect directly to an established osteoporosis pathway (11, 14). Sagittal CT reformations should be reviewed for vertebral morphology and fracture reporting (14). Fracture severity can be graded using the Genant semiquantitative method, which classifies vertebrae as normal or as mild, moderate, or severe fracture (grades 0−3) using morphology and height loss (22). The report should specify vertebral level and severity and should consider trauma, malignancy, infection, congenital variation, Scheuermann-type or degenerative change, and other mimics before attributing a deformity to osteoporosis (11, 20, 22).

Retrospective studies have combined CT biomarkers with demographic or clinical variables for fracture-risk prediction (57–59). Dagan et al. combined vertebral fracture, simulated DXA T-scores, lumbar trabecular density, age, and sex and reported a modest improvement in major osteoporotic fracture prediction compared with the Fracture Risk Assessment Tool without bone mineral density (57). Automated abdominal CT biomarkers predicted future major osteoporotic fractures in an asymptomatic screening cohort (58). In a separate matched case-control study, lower L1 attenuation, CT-derived femoral-neck T-scores, and prevalent vertebral fractures were associated with future hip fracture (59). These studies support retrospective risk enrichment but do not establish clinical net benefit or the effectiveness of CT-triggered intervention, and related institutional screening cohorts should not be treated as fully independent replication.

Implementation evidence is beginning to extend beyond retrospective detection. Dogra et al. described an early clinical osteoporosis-screening program in which automated vertebral measurements are presented in a picture archiving and communication system (PACS), verified by the interpreting radiologist, routed through the radiology report and electronic health record, and linked to a dedicated bone-health pathway for confirmatory DXA and follow-up (9). The authors also stated that the program was in an early stage and that long-term outcomes were not yet available (9). Thus, implementation experience supports feasibility and workflow design but does not yet establish universal patient benefit.

## 4 Muscle compartment: quantity, quality, and sarcopenia risk

### 4.1 Muscle quantity

CT can provide reproducible estimates of skeletal muscle cross-sectional area when anatomic level, segmentation, acquisition, and normalization are standardized (2, 6, 7, 24). Cross-sectional area should be described as a surrogate for muscle quantity rather than as muscle mass itself (23, 24, 30). Foundational single-slice studies linked standardized abdominal measurements with whole-body muscle or body-composition measures (23, 24), whereas more recent artificial intelligence (AI) studies have added internal, external, and dual-center validation of automated muscle and adipose segmentation (6, 7). A prospective multicenter PACS-integrated study further demonstrated rapid automated L3 selection and body-composition analysis in routine health-check CT, supporting workflow feasibility while not by itself demonstrating improved patient outcomes (10).

For complete L3 analysis, the intended muscle compartment should be segmented consistently and should include the prespecified psoas, paraspinal, rectus abdominis, transversus abdominis, quadratus lumborum, and internal and external oblique muscles when those structures are part of the chosen method (6). Organ tissue, bowel, kidney, and adipose tissue should not be included as skeletal muscle, and target abdominal-wall or posterior muscle groups should not be omitted (6, 7). The measured muscle group, vertebral level, units, attenuation range if used, segmentation boundaries, and normalization method should be stated explicitly (2, 6, 25). Psoas-only and masseter measures have been used in specific settings (41, 42); because they quantify different anatomic compartments, they should be reported separately from complete L3 muscle analysis (2).

In oncology, the prognostic meaning of CT muscle measurements depends on cancer type, stage, treatment, timing, and the threshold applied (34–38). Low skeletal muscle reserve and sarcopenic-obesity patterns have been associated with adverse outcomes in selected tumor cohorts (34, 35), while treatment-related muscle loss may carry different information from a baseline threshold (36). Cohort-derived cutoffs incorporated into prognostic models should therefore be reported as disease- and method-specific risk thresholds rather than as general diagnostic criteria for sarcopenia (37).

Outside oncology, single-muscle measures have been associated with outcomes in older trauma and advanced liver disease: masseter cross-sectional area in older trauma (41) and psoas-based measurements in cirrhosis (42). Complete L3 skeletal muscle index has also been evaluated in end-stage liver disease (43). Because these approaches quantify different anatomic compartments, they should be reported separately rather than treated as interchangeable thresholds (2, 41–43). Cachexia requires weight trajectory and other clinical domains (39, 40), whereas sarcopenia requires the multidomain criteria specified by the applicable consensus framework, combining muscle quantity with muscle strength and/or physical performance as applicable (45, 46). CT can contribute to either body-composition phenotype but does not establish the clinical diagnosis by itself. Conventional nutrition-screening classifications and CT-defined muscle abnormalities are related but nonidentical and can be discordant in oncology (38); such discordance does not establish that conventional screening generally fails, and malnutrition should not be inferred from a CT muscle measurement alone.

### 4.2 Muscle quality, low muscle attenuation, and terminology

Mean muscle attenuation in HU is widely used as an imaging surrogate related to tissue composition because lower attenuation is generally associated with greater intramuscular lipid, but it is not a direct measurement of histologic fat, strength, or function (25). Contrast phase is an important source of variability (31–33). In Boutin et al., both psoas and posterior paraspinal muscle attenuation increased significantly during arterial, portal, and delayed phases, with the greatest mean enhancement in the delayed phase (approximately 13 HU); mean enhancement was similar between the two muscle groups, but associations with age, sex, and baseline attenuation differed by region (31). In an automated multiphase study, mean muscle density increased from 40.0 HU before contrast to 51.5 HU in the portal venous phase and 54.2 HU in the delayed phase, whereas skeletal muscle area was much less affected (33). A 459-patient multiphase study similarly showed that fixed cutoffs can cause phase-related reclassification, particularly for low muscle attenuation (32). Contrast phase should therefore be reported, and longitudinal comparisons should preferentially use comparable acquisition phases (31–33).

Dual-energy CT can estimate skeletal muscle fat fraction through material decomposition or related post-processing methods (26). In a small study comparing dual-energy CT with magnetic resonance imaging, dual-energy CT-derived muscle fat fraction correlated with 3-T magnetic resonance imaging fat fraction, supporting technical feasibility but not a mature diagnostic threshold (26). Such methods remain acquisition- and post-processing-dependent (25, 26). When only CT radiodensity has been measured, this review uses the descriptive term “low muscle attenuation”; the term myosteatosis should be used only when the operational definition, acquisition context, and threshold are stated.

Terminology should reflect the clinical consensus being applied. The revised European Working Group on Sarcopenia in Older People consensus centers case identification on low strength and requires low muscle quantity or quality for confirmation, whereas AWGS 2019 and the 2025 AWGS update use related but distinct criteria (44–46). The 2022 ESPEN/EASO consensus on sarcopenic obesity also requires muscle function and body-composition assessment rather than a CT-only label (47). CT may describe muscle area, skeletal muscle index, or attenuation, but these measurements do not establish sarcopenia without the applicable clinical criteria (44–46). Any suggestion to correlate such findings with strength, nutrition, physical function, frailty, or rehabilitation needs is presented here as illustrative author-proposed reporting language rather than as a consensus-mandated CT reporting rule. Table 2 therefore provides illustrative, author-proposed wording rather than validated reporting guidelines.

**Table 2:** Illustrative author-proposed reporting language for opportunistic CT findings (not a validated guideline).

Finding	Suggested wording	Avoid	Possible clinical correlation
Low vertebral attenuation	“Low trabecular attenuation at [level] on this examination, which may indicate a higher probability of low bone mass” (8, 12, 13, 15–21, 31).	“CT-diagnosed osteoporosis” based on HU alone.	Consider DXA or clinical fracture-risk assessment according to patient context and local pathway; document level, HU, kVp, contrast phase/status, reconstruction, and method.
Confirmed vertebral fracture	“Vertebral fracture at [level], visually graded as [mild/moderate/severe]; correlate with trauma history and non-osteoporotic causes. If atraumatic or low-trauma, osteoporosis assessment may be appropriate” (11, 14, 22).	Calling every vertebral deformity an osteoporotic or compression fracture without visual confirmation.	Differentiate trauma, tumor, infection, congenital and degenerative mimics; follow the applicable fracture pathway.
Low muscle area or skeletal muscle index	“Skeletal muscle cross-sectional area is low relative to the stated disease- or population-specific reference” (2, 6, 7, 24).	“Low muscle mass” as a direct CT measurement, or “sarcopenia” without application of the relevant consensus clinical and functional criteria.	Correlate with strength, nutrition, physical function, frailty, symptoms, and rehabilitation needs.
Low muscle attenuation	“Low muscle attenuation on this examination; interpretation is protocol-, contrast-phase-, and clinical-context dependent” (25, 31–33).	Direct claims about strength/function or unqualified diagnosis of myosteatosis.	Review contrast phase and reconstruction; interpret with muscle quantity and clinical status.
High VAT or adverse distribution	“Increased visceral adipose tissue relative to the stated measurement method or reference” (27, 28).	“CT-diagnosed metabolic syndrome.”	Integrate with body size and cardiometabolic risk factors when clinically relevant; this is not a CT-only diagnosis of metabolic syndrome.
Low or declining SAT	“Low or declining subcutaneous adipose tissue on technically comparable examinations” (27, 40, 62).	“CT-diagnosed cachexia” from one scan.	Review weight trajectory, intake, disease status, inflammation, and overall nutrition when clinically relevant.
Combined abnormal pattern	“Combined abnormalities may involve bone-muscle patterns (30, 49) or muscle-adipose patterns (47); each component requires separate interpretation.”	A universal CT-only osteosarcopenic-obesity, sarcopenic-obesity, or triad diagnosis.	Treat the combined pattern as hypothesis-generating and translate each component into an established or locally validated clinical assessment.

## 5 Adipose compartment: VAT, SAT, and related CT measures

### 5.1 CT quantification of adipose compartments

CT can distinguish VAT from SAT at standardized abdominal levels when the complete body contour is included (6, 7, 24, 27). Foundational single-slice studies established associations between regional measurements and whole-body adipose volumes (23, 24), whereas later automated studies demonstrated high-throughput segmentation of VAT and SAT with internal, external, and dual-center evaluation (6, 7, 27). A prospective multicenter PACS-integrated system further demonstrated automated L3 selection and body-composition analysis in routine health-check CT, supporting technical workflow feasibility (10).

VAT area and SAT area quantify distinct anatomic compartments, whereas the VAT/SAT ratio is a relative distribution metric (27, 28). Adipose attenuation is a separate radiodensity measure (48), and longitudinal fat loss describes change over time rather than a baseline phenotype (27). In a large asymptomatic screening cohort, the VAT/SAT ratio was associated with subsequent cardiovascular events and mortality, but this was a cohort-specific risk association rather than a universal CT definition of metabolic disease (28).

Related CT studies provide cardiometabolic context for visceral adiposity. In 208 subjects with clinical suspicion of coronary artery disease, Wu et al. found abdominal visceral adipose tissue volume to be independently associated with metabolic syndrome, whereas regional epicardial adiposity was more closely associated with coronary atherosclerotic burden, supporting the concept that different adipose depots may carry distinct cardiometabolic information (60). An earlier report from the same group evaluated left atrioventricular-groove epicardial adipose tissue thickness, a cardiac adipose measure outside the abdominal VAT/SAT focus of this review, and found that adding it to clinical risk factors and Agatston score improved discrimination for obstructive coronary artery disease (area under the curve, 0.848 to 0.912) (61). The 2016 report used the same 208-subject institutional cohort as the 2013 study and therefore should not be regarded as independent replication (60, 61). These retrospective findings provide adjacent cardiometabolic context but do not establish universal screening thresholds for abdominal VAT/SAT measures.

### 5.2 Technical and clinical caveats

Adipose measurements depend on consistent anatomic level selection, attenuation ranges, segmentation method, and complete inclusion of the body contour (6, 7, 27). Failure modes documented in automated or implementation studies include subcutaneous edema or ascites, metallic artifacts, inaccurate level selection, motion degradation, and insufficient coverage (6, 7, 9). Contrast administration also changes adipose measurements (32, 48). In a multiphase study of VAT and SAT, Gohmann et al. found that iodinated contrast generally increased adipose density and decreased segmented area, with larger effects in later phases and in VAT than in SAT; the maximum reported density increase was approximately 5.6 HU and area reductions approached 11% in some comparisons (48). Vedder et al. also found statistically significant phase-related changes in VAT area together with muscle measurements (32). Contrast phase should therefore be recorded when adipose attenuation, fixed thresholds, or longitudinal change are interpreted (32, 48).

A single CT examination should not be used as a standalone diagnosis of cachexia (39, 40) or sarcopenic obesity (47). In the Wisconsin screening cohort, VAT/SAT ratio was evaluated as a cohort-specific risk biomarker rather than as a CT diagnostic definition of metabolic syndrome (28). CT-defined abnormal body-composition phenotypes may also be discordant with conventional nutrition-screening classifications in oncology (38). Excess VAT may be reported as contextual body-composition information (27, 28). Low or declining adipose stores should be interpreted with weight trajectory, intake, inflammation, disease status, and function when cachexia is suspected (39, 40); when change is reported from serial CT, scan interval and technical comparability should also be described (62). Fixed thresholds should not be transferred across sex, age, body size, disease, acquisition phase, or measurement level without validation (27, 32, 48). The principal anatomical targets and protocol-dependent sources of variation across the three tissue compartments are summarized in Figure 2.

**Figure 2:**
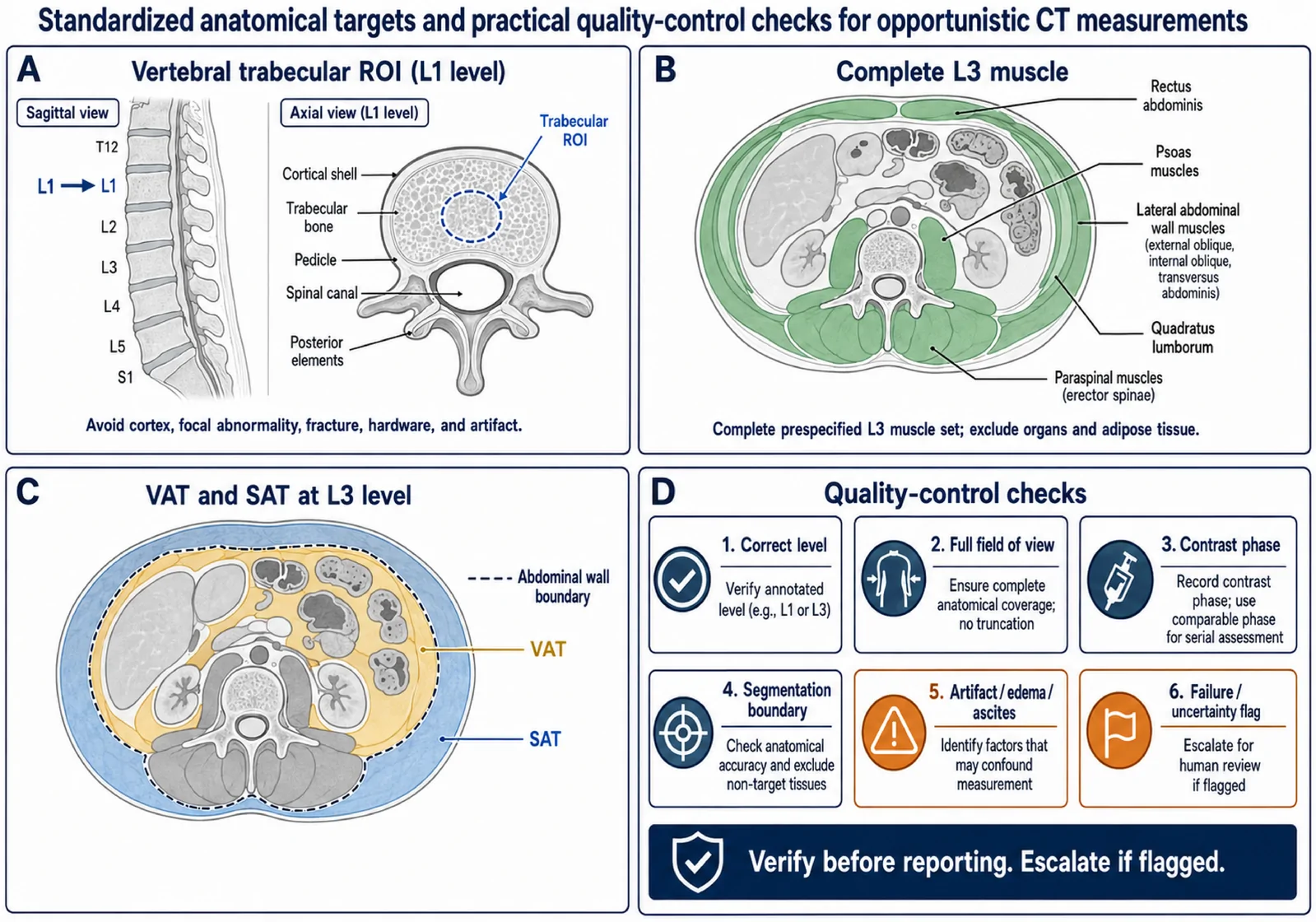
Schematic illustration of standardized anatomical targets and practical quality-control checks for opportunistic CT measurements. The anatomical drawings are conceptual illustrations intended to demonstrate measurement targets and quality-control principles; they are not clinical segmentation outputs or examples of algorithm-validation performance. **(A)** Manual vertebral trabecular attenuation uses an intratrabecular ROI that avoids cortex, focal abnormality, fracture, hardware, and artifact (16); automated 3D placement follows algorithm-specific rules (8). **(B)** Complete L3 muscle assessment should follow a predefined muscle-group convention and exclude kidneys, other organs, and adipose tissue (6, 7). **(C)** VAT and SAT should be separated relative to the abdominal muscular wall (6, 27, 48). **(D)** The displayed quality-control checks are an author-proposed practical checklist informed by automated and implementation studies and include correct anatomic level, complete field of view, contrast phase, segmentation boundaries, artifacts or edema/ascites, and failure or uncertainty flags (6, 7, 9). Flagged or anatomically implausible results should undergo human review before reporting.

## 6 Integrated assessment of bone, muscle, and adipose tissue

### 6.1 Clinical rationale and limits of combined assessment

Osteosarcopenia literature provides a bone-muscle rationale for combined assessment (49), while sarcopenic-obesity guidance supports integrated interpretation of muscle and adiposity (47); the 2025 AWGS update emphasizes skeletal-muscle cross-talk with bone and adipose tissue (44). This biological rationale does not itself establish incremental value for a combined CT model. A 2026 integrated CT study showed technically accurate simultaneous L3 muscle and vertebral segmentation and cross-sectional associations with bioelectrical impedance analysis-derived muscle indices, functional impairment, and DXA-derived T-scores, but did not test a validated three-compartment predictive or treatment pathway and called for external and prospective validation (30). The three-compartment framework proposed in this review is therefore hypothesis-generating rather than a validated syndrome or clinical score. The integrated and longitudinal interpretation framework is illustrated in Figure 3.

**Figure 3:**
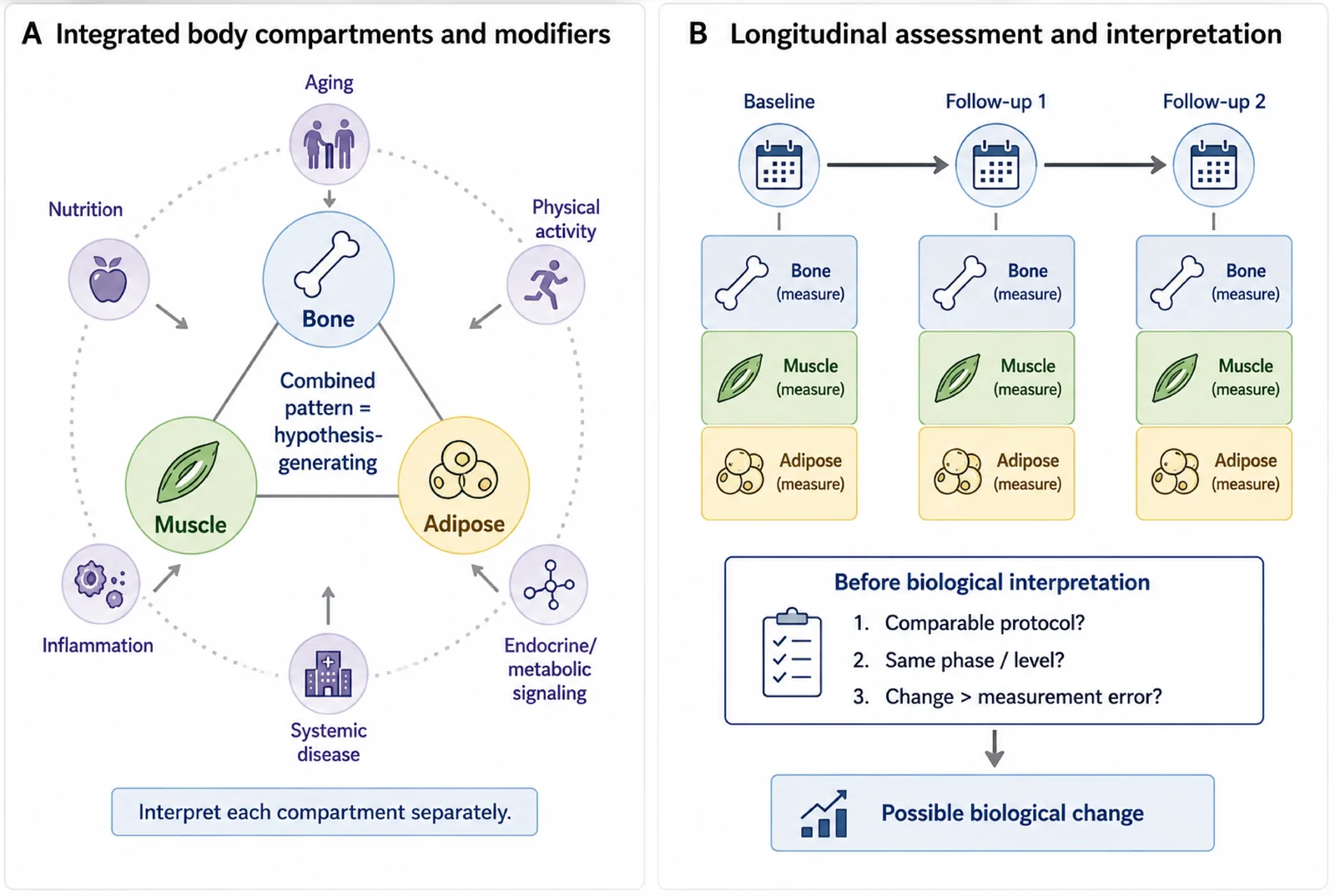
Integrated and longitudinal interpretation of opportunistic CT findings. **(A)** Osteosarcopenia literature provides a biological rationale for bone-muscle interaction (49), and sarcopenic-obesity guidance provides a rationale for integrated interpretation of muscle and adiposity (47). The three-compartment framework shown here is author-proposed and hypothesis-generating; it does not represent a validated combined diagnostic syndrome or clinical score because current integrated CT evidence remains predominantly cross-sectional (30). Each compartment should therefore be interpreted separately. **(B)** Serial change should be interpreted only after confirming comparable acquisition and analysis conditions and considering measurement precision and a method-specific least significant or minimum detectable change (62, 63). A change exceeding measurement error may represent biological change but does not by itself establish disease progression or treatment response.

Sarcopenic obesity is discussed here, rather than in the technical adipose-quantification section, because it is a clinical syndrome requiring integrated assessment. The 2022 ESPEN/EASO consensus defines sarcopenic obesity as the coexistence of excess adiposity and low muscle mass/function and proposes a diagnostic pathway that begins with skeletal muscle function and then confirms altered body composition (47). CT may contribute information on muscle quantity and adipose distribution, but a low-muscle/high-adiposity CT pattern alone does not satisfy that diagnostic pathway (47). Similarly, osteoporosis is assessed using fracture history, DXA, and clinical criteria (11), sarcopenia requires the multidomain criteria specified by the applicable consensus framework (44–46), and cachexia requires clinical and longitudinal information beyond CT body composition (39, 40). Reports are therefore more defensible when each abnormal compartment is described separately and linked to appropriate clinical correlation.

### 6.2 Longitudinal trajectories

Serial CT can show body-composition trajectories in patients who already undergo repeated imaging. Treatment-related muscle loss has been associated with survival in selected oncology cohorts (36), and automated studies have demonstrated longitudinal measurement of VAT, SAT, muscle area, and attenuation (27, 29). Lee et al. (27), Pickhardt et al. (28), and Graffy et al. (29) arose from related Wisconsin CT-colonography screening cohorts and should be regarded as related rather than fully independent replication. These studies establish feasibility and selected prognostic associations, not universal thresholds for clinically meaningful change. Evidence for serial vertebral attenuation as a treatment-response biomarker remains particularly limited.

Technical comparability is necessary but not sufficient for longitudinal interpretation. Vertebral attenuation changes with contrast phase and protocol (19, 21, 31). Muscle attenuation is contrast-phase sensitive (25, 31–33), and adipose measurements also change with contrast phase and segmentation method (32, 48). Subcutaneous edema or ascites, metallic artifacts, incorrect level selection, and incomplete or motion-degraded studies can compromise automated body-composition segmentation or interpretation (6, 7, 9). Precision testing is therefore needed to distinguish measurement error from biological change (62, 63). In 38 patients with head-and-neck cancer, Arribas et al. reported same-image intra-observer precision errors of 0.26%–0.86% and corresponding 95% least significant change values of 0.7%–2.4% across thigh, chest, abdomen, and upper arm measurements, illustrating that detectable change is method- and site-specific (63). A scoping review found that measurement error was discussed in only 8 of 34 cohorts and recommended explicit reporting of scan interval and attention to measurement error (62). Before serial differences are labeled progression or treatment response, they should exceed an appropriate method-specific error threshold and the derivation of that threshold should be stated (62, 63).

### 6.3 Automation, standardization, and quality control

Manual measurements remain valuable for validation but are difficult to incorporate into high-volume practice (3, 5). Park et al. reported high internal and external performance for automated L3 muscle, VAT, and SAT segmentation (6), and Nowak et al. combined automated level selection, tissue segmentation, and integrated quality control with dual-center end-to-end evaluation (7). Urooj et al. prospectively implemented a PACS-integrated automated body-composition system across multiple centers and reported rapid processing and high segmentation accuracy without manual adjustment in their study workflow (10). Bone-focused automation has also scaled to very large multi-scanner datasets (8), while Dogra et al. described an early radiologist-verified clinical osteoporosis program with explicit downstream care responsibilities (9). These studies represent progressively stronger technical and implementation evidence, but none alone proves improved patient outcomes from routine reporting.

Standardization remains a major barrier across tissues. For bone, age and ROI placement affect vertebral attenuation (16), tube voltage and scanner model can shift HU values (8), contrast phase can alter attenuation and classification (19, 21, 31), and calibration/phantomless methods introduce additional method dependence (20). For muscle, attenuation is acquisition- and contrast-phase sensitive (25, 31–33). Automated muscle and adipose measurements also depend on level selection and segmentation quality (6, 7), while adipose attenuation and segmented area can shift with contrast phase and segmentation method (32, 48). Earlier dual-layer spectral CT scout work demonstrated technical feasibility for DXA-like areal bone mineral density estimation (64), while photon-counting detector CT now provides a current spectral platform for similar opportunistic approaches (65). In a prospective study of 51 participants, photon-counting detector CT spectral localizer-derived T-scores showed a Lin concordance correlation coefficient of 0.90 with DXA-derived T-scores and high sensitivity for abnormal bone mass, but the small cohort and platform-specific method support an emerging rather than established role (65).

Human quality control should be explicit and efficient. Automated pipelines can flag low-confidence or technically unsuitable studies, and published workflows keep the selected level and segmentation overlay reviewable (6, 7, 9). Published segmentation studies document problems such as edema/ascites and metallic artifacts (6, 7), and implementation work excludes or flags motion-degraded scans, insufficient coverage, or hardware artifact (9). Building on those observations, this review proposes that the reviewer should also examine major deformity, tumor, or surgery when these conditions could invalidate the measurement. As a practical author-proposed quality-control checklist, the reviewer should confirm the intended CT series and contrast phase, correct anatomic level, complete field of view, anatomically plausible segmentation boundaries, absence of organ inclusion or missing target muscle groups, correct VAT/SAT separation relative to the abdominal wall, and the absence of artifacts or conditions that invalidate interpretation. Quantitative values, uncertainty indicators, and failure flags should also be checked for plausibility (7, 9). An exception-based workflow, in which detailed review is triggered by failure or low-confidence flags, truncation, implants, anatomic distortion, or implausible values, is consistent with the quality-control strategies described in automated pipelines and implementation frameworks (7, 9).

An illustrative author-proposed staged approach, informed by implementation and prospective workflow literature, is to move from retrospective local validation to prospective shadow mode, then a limited structured-reporting pilot, and only then routine use after thresholds, quality-control rules, software governance, and follow-up responsibilities are agreed (5, 9, 10). The pathway in Table 3 is illustrative and author proposed, not a validated guideline. Before deployment, the program should name who verifies the measurement, who incorporates or communicates the finding, who orders or activates confirmatory assessment, who closes the follow-up loop, and who oversees software performance and drift (9). As an author-proposed monitoring set informed by expert-panel and implementation concerns, programs should track workload, false-positive downstream testing, alert frequency and fatigue, follow-up completion, unequal access to confirmatory care, subgroup performance, and model or threshold drift (5, 9).

**Table 3:** Illustrative author-proposed stages for implementing an opportunistic CT program (not a validated guideline).

Stage	Minimum requirements	Quality control and monitoring	Responsibility
1. Retrospective local validation	Define intended use, eligible protocols, reference standard, exclusions, local thresholds, and failure criteria (5, 9); where longitudinal use is intended, define repeatability and detectable change (62, 63).	Compare automated and manual/reference measurements; analyze failure modes, calibration, subgroup performance, and detectable change where longitudinal use is intended.	Local radiology, imaging science, statistics, and relevant clinical services jointly approve the intended use.
2. Prospective shadow mode	Display the measurement, anatomic level, overlay, acquisition context, and uncertainty/failure information (7, 9, 10); define human verification and routing before autonomous patient-facing labeling (9).	Prospectively measure agreement, false positives, failure rates, alert frequency, radiologist review time, and alert fatigue.	A radiologist or designated imaging professional verifies output; a named technical owner monitors software performance.
3. Limited structured-report pilot	Use conservative, illustrative wording and a predefined confirmation/referral pathway in a restricted service line (5, 9).	Track clinician acceptance, confirmatory testing, follow-up completion, patient communication, workload, false-positive testing, and unequal access to follow-up.	A named clinical service receives findings and orders/activates confirmatory assessment; a designated coordinator or service closes the follow-up loop.
4. Routine clinical program	Integrate routing, communication, confirmatory assessment, and software oversight (9); establish local registry/audit functions and escalation policies as governance measures; review reimbursement and coding locally (5, 9).	Audit outcomes, workload, alert burden, subgroup performance, equity, calibration, software/model drift, threshold drift, and downstream testing.	Joint governance assigns explicit clinical, technical, operational, legal, information-governance, and follow-up accountability.

## 7 Clinical implementation and governance

### 7.1 Linking imaging findings to clinical follow-up

Osteoporosis provides the most direct follow-up pathway among the applications reviewed (5, 9, 11, 12). Low vertebral attenuation can support consideration of DXA or clinical fracture-risk assessment within a protocol-validated program, whereas a visually confirmed vertebral fracture compatible with fragility fracture may constitute a clinically consequential fracture history and can trigger an established osteoporosis pathway (11, 13, 14, 22). These findings should not be treated as equivalent triggers. The effect of reporting either finding depends on reliable communication, confirmation, and closed-loop follow-up, as emphasized in implementation literature (9).

Muscle and adipose findings require pathways tailored to the clinical setting. Disease-specific CT muscle associations have been reported in oncology (35–38), older trauma (41), and advanced liver disease (42, 43), and treatment-related muscle change can be prognostic in selected oncology cohorts (36). These studies do not supply a universal CT threshold for sarcopenia; consensus frameworks require assessment beyond a CT muscle threshold and incorporate muscle quantity, muscle strength, and/or physical performance according to the applicable framework (45, 46). Depending on context, follow-up may focus on strength and function for sarcopenia (44–46), weight, intake, and disease trajectory for cachexia (39, 40), or combined muscle-function and body-composition assessment for sarcopenic obesity (47). The measurement method, reference population, contrast phase, uncertainty, and intended clinical question should be stated (25, 31–33, 48).

Hybrid imaging provides another opportunity only when the CT component is technically suitable. As a conservative implementation rule, low-dose localization or attenuation-correction CT from hybrid examinations should not be assumed equivalent to diagnostic-quality body CT unless the intended biomarker has been protocol-specifically validated. For attenuation-based bone, muscle, and adipose measures, acquisition factors such as tube potential, reconstruction, contrast phase, and anatomic coverage can affect interpretation (2, 5, 8, 19, 25, 31–33, 48). The specific parameters relevant to the intended biomarker should therefore be documented and locally validated rather than presumed interchangeable.

### 7.2 Patient communication, equity, and responsibility

Patient communication should distinguish an imaging biomarker abnormality from a confirmed diagnosis (5, 9). A practical message is that an existing CT examination has shown a bone, muscle, or adipose finding that may warrant clinical correlation or further assessment; the CT measurement alone has not established a new disease (11, 45–47). This is consistent with a risk-enrichment and triage role rather than standalone diagnostic use (3, 5, 9). Patient understanding, acceptance, and communication methods remain important outcomes for prospective implementation evaluation (5, 9).

Because opportunistic assessment extends beyond the original imaging question, institutions should define eligible examinations, intended use, thresholds, verification, documentation, result routing, patient communication, data retention, software status, information security, confirmatory testing, and follow-up responsibility before routine deployment (5, 9). The workflow should explicitly name the imaging professional who verifies the measurement, the clinician or service that receives and communicates it, the party authorized to order or activate confirmatory assessment, and the person or service responsible for documenting completed follow-up (9). Workload, false-positive downstream testing, alert fatigue, software or threshold drift, and unequal access to follow-up should be monitored as implementation outcomes (5, 9). An illustrative closed-loop implementation pathway is shown in Figure 4.

**Figure 4:**
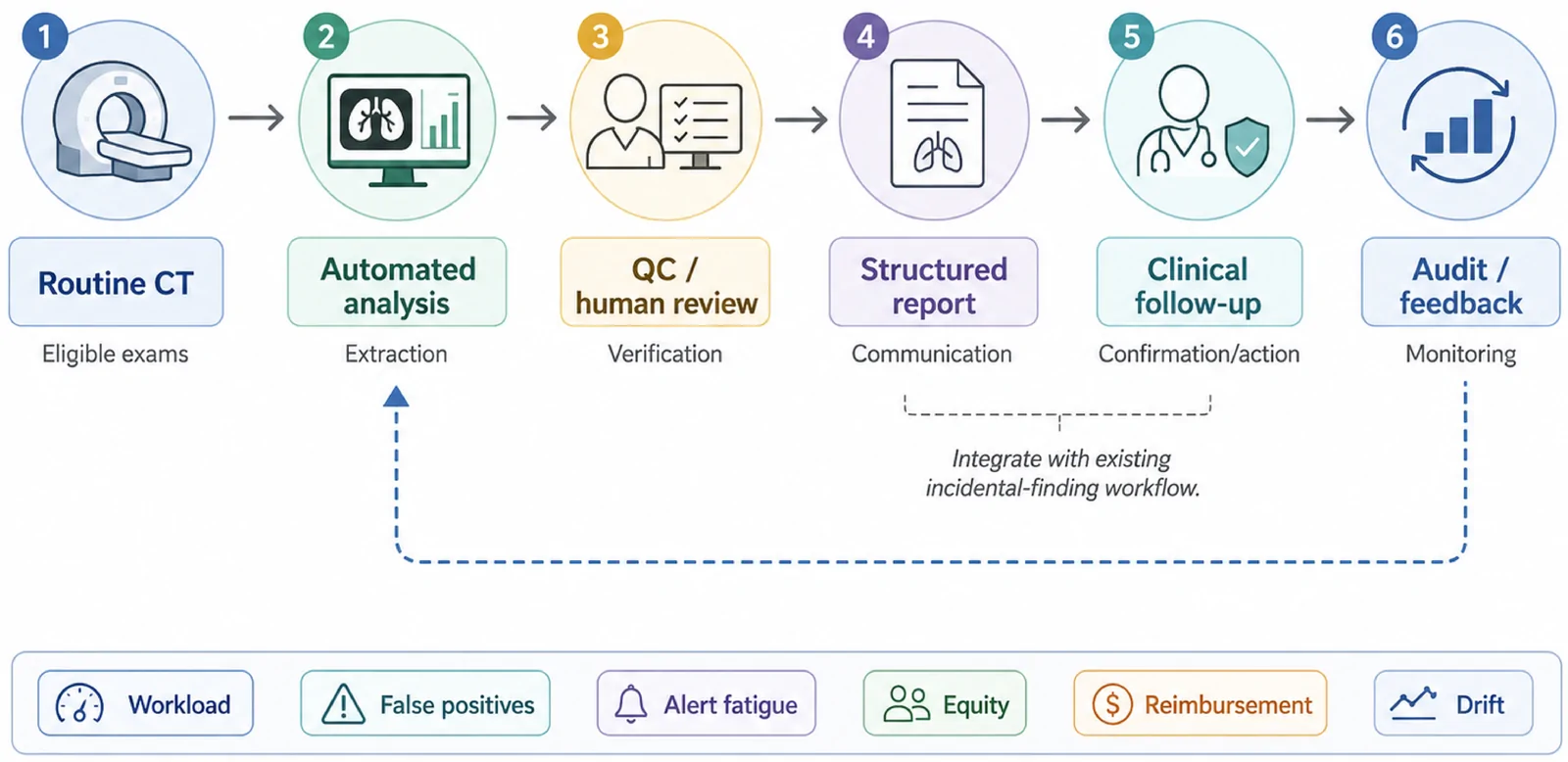
Illustrative closed-loop implementation pathway for opportunistic CT biomarkers. The workflow is an author-proposed synthesis informed by opportunistic-screening implementation literature and emphasizes automated analysis, human verification, structured reporting, defined clinical follow-up, and audit/feedback (5, 9). Before implementation, institutions should explicitly assign responsibility for measurement verification, reporting and communication, confirmatory assessment, follow-up completion, and technical oversight. Opportunistic results should be integrated with existing incidental-finding workflows to reduce unnecessary or duplicative downstream actions (67). As an author-proposed monitoring set informed by expert-panel and implementation concerns, programs should track workload, false-positive testing, alert burden or fatigue, equity, reimbursement, and software or threshold drift (5, 9). The pathway is illustrative and is not a validated guideline.

Reimbursement and coding are jurisdiction-specific barriers rather than settled components of opportunistic CT practice (5, 9). The Radiology Scientific Expert Panel described the United States Current Procedural Terminology category framework and noted that a Category III pathway has been used for vertebral-fracture detection, while emphasizing that most Category III services are not routinely reimbursed by Medicare or other United States insurers (5). Dogra et al. likewise noted that reimbursement pathways for AI radiology tools remain limited and that early programs may depend on institutional support or grants (9). A published Markov cost-effectiveness model suggested that AI-assisted opportunistic CT screening for cardiovascular disease, osteoporosis, and sarcopenia could be cost-saving under a range of modeled assumptions, but this was a hypothetical-model analysis rather than evidence of real-world patient benefit (66). No universal billing code should therefore be assumed for opportunistic bone, muscle, or adipose interpretation; local coding, payer, and health-system expertise is required before deployment.

The American College of Radiology incidental-findings white paper highlights the potential harms and costs of unnecessary diagnostic cascades and the need for standardized management of incidental findings (67). Opportunistic body-composition assessment should therefore be integrated with existing result-management policies rather than operate as a parallel alert system, consistent with current opportunistic-screening implementation frameworks (5, 9). A single CT may generate multiple findings; as an author-proposed governance measure, institutions should define prioritization, deduplication, communication channels, and escalation rules to avoid competing alerts and unnecessary downstream testing (5, 9).

Large-scale CT data show that vertebral attenuation varies across age, sex, race and ethnicity, tube voltage, and scanner model (8). Expert and implementation literature therefore emphasizes validation in the intended population and continued performance monitoring after deployment (5, 9). Equity assessment should extend beyond algorithm accuracy to downstream access and follow-up completion; this is an implementation outcome proposed for prospective evaluation (5, 9).

## 8 Discussion

Opportunistic CT can extract clinically relevant quantitative information from examinations performed for other reasons, but the evidence hierarchy is uneven (1–5, 12). Vertebral fracture detection and protocol-specific vertebral attenuation have the most direct clinical pathway because fracture history and osteoporosis assessment are already embedded in established care (9, 11, 12, 14, 22). Technical validation of L3 CT muscle measurement and automation is substantial (2, 6, 7, 10, 24), while foundational single-slice rationale also includes modality-independent body-composition work (23). Disease-specific prognostic or nutritional associations are reported in selected oncology, trauma, and liver-disease cohorts (34–38, 41–43). Adipose and longitudinal studies in this review are largely technical, observational, or methodological (27, 28, 48, 62, 63); the integrated CT study is cross-sectional (30), and the prospective automated workflow study reports technical and implementation outcomes rather than patient outcomes (10). Accordingly, demonstrated patient-benefit evidence remains limited. Technical feasibility, clinical association, prediction, clinical utility, and patient benefit should therefore remain distinct claims.

The evidence base has important methodological limitations. Many studies are retrospective, single-center, disease-specific, or derived from screening cohorts; thresholds are often internally derived; protocols and contrast phases vary; and publications from related institutions may involve overlapping or non-independent cohorts (12, 17, 18, 27–29, 32, 62). Large-scale automation, external validation, prospective workflow evaluation, and clinical implementation frameworks strengthen parts of the literature (6–10); in this review's synthesis, they do not remove the need for independent replication and outcome-based evaluation. Longitudinal interpretation is particularly vulnerable to measurement error, and method-specific repeatability or least significant change should precede claims of biological progression or treatment response (62, 63).

This review is itself limited by its narrative design. The search process did not retain a screening log, study selection was not performed in duplicate, and no formal design-specific risk-of-bias assessment or quantitative synthesis was undertaken. The search and evidence framework improve transparency but do not make the review systematic. Based on the gaps synthesized above, future studies should prioritize prospective clinical-utility and patient-benefit outcomes, including referral completion, DXA uptake, treatment or supportive-care actions, fractures, physical function, nutritional outcomes, patient understanding, false-positive testing, workload, alert fatigue, subgroup performance, equity, software drift, and cost. Until such evidence is available, local validation, conservative terminology, visible quality control, and closed-loop responsibility are preferable to universal diagnostic or implementation claims (5, 9).

## 9 Conclusion

Routine CT can provide opportunistic information about bone, skeletal muscle, and adipose tissue when the existing examination is technically suitable (1–5). The evidence is strongest for visually confirmed vertebral fracture and protocol-specific vertebral attenuation as pathways to established osteoporosis assessment (9, 11, 12, 14, 22). CT muscle cross-sectional area is a surrogate for muscle quantity (24), while muscle attenuation is a protocol-sensitive tissue-composition measure (25). Neither alone establishes sarcopenia under consensus criteria (44–46). Adipose and serial-change evidence remains largely technical or observational (27, 28, 48, 62, 63), combined assessment remains cross-sectional (30), and prospective automated work has primarily demonstrated workflow feasibility (10); these sources do not establish broad patient benefit. Biological pairwise interactions are described in sarcopenic-obesity and osteosarcopenia literature (47, 49), whereas available integrated CT evidence remains cross-sectional (30); therefore the three-compartment framework proposed here should currently be regarded as hypothesis-generating rather than a validated integrated bone-muscle-adipose model. Safe translation requires protocol-aware measurement, practical human quality control, explicit communication and follow-up responsibility, integration with incidental-findings workflows, governance, and outcome audit (5, 9).
